# A qualitative study of smoking behavior among the floating population in Shanghai, China

**DOI:** 10.1186/1471-2458-14-1138

**Published:** 2014-11-04

**Authors:** Ji-Wei Wang, Zhi-Ting Cui, Ning Ding, Cheng-Gang Zhang, Tricia Usagawa, Helen Louise Berry, Jin-Ming Yu, Shen-Sheng Li

**Affiliations:** Key Laboratory of the Public Health Safety, Ministry of Education, Department of Health Behavior and Health Education, School of Public Health, Fudan University, Shanghai, China; Health Bureau of Xuhui District, Shanghai, China; Faculty of Health, The University of Canberra, Canberra, ACT Australia; Xuhui Center for Disease Prevention and Control, Shanghai, China; Department of Native Hawaiian Health, University of Hawaii at Manoa, Honolulu, USA

**Keywords:** Floating population, Smoking, China, Qualitative study

## Abstract

**Background:**

China has become the world’s largest producer and consumer of tobacco and lung cancer is China’s leading cause of cancer deaths. The large majority of Chinese smokers are men. Tobacco consumption is of particular concern among China’s internal floating (or migrant) population, which has become a permanent feature of Chinese society, because this population is very large (over 100 million persons) and it has a high prevalence of smoking. Considering additionally that like the general population of China, the smoking prevalence rate of women from this group is quite low, we therefore aimed to explore smoking-related knowledge, attitudes and behaviours among male smokers in the floating population to help inform the development of effective smoking cessation interventions in this important target group in China.

**Methods:**

We interviewed 39 floating population male smokers in six focus groups and performed a qualitative content analysis of the interviews.

**Results:**

Most participants knew that smoking is risky to health but they knew little about why. Habit and social participation were key drivers of smoking. Smoking was regarded as a core component of their identity by the urban residents. Some participants had tried to stop smoking but none reported having ever been educated about smoking.

**Conclusions:**

Smoking cessation interventions for China’s male floating population would need to incorporate comprehensive education and information about why smoking is dangerous and the benefits of stopping.

## Background

In 2010, there were an estimated 301 million current tobacco smokers in China, accounting for 28.1% of the adult population (52.9% of men and 2.4% of women), making China the largest tobacco consumer in the world [1]. Smoking contributes to four of China’s five leading causes of death and about 3,000 people die every day in China due to smoking [2]. Those living with disadvantage are more likely to smoke and less likely to stop smoking than are their non-disadvantaged peers [3, 4]. Smoking is not only patterned by social inequalities, but is also a major contributor to inequalities in health [5]. In 2003, China’s National People’s Congress announced its decision to ratify the World Health Organization Framework Convention on Tobacco Control [6]. Nevertheless, there remain overwhelming barriers to implementing effective tobacco control policies and programs, one of which is related to the large scale of China’s internal floating (or migrant) population which is characterised by substantial disadvantage.

The floating population grew rapidly following China’s 1979 program of economic reform which created abundant employment opportunities in urban areas and relaxed regulations restricting population movements. As a result, a large and growing number of rural people moved from the central and western parts of China to the eastern and coastal urban areas in pursuit of higher incomes [7]. In 2000, it was already estimated at 121 million, representing 10% of China’s total population at that time [8], with 83% of working-age (15–64 years) and 52% men. In 2011 the number reached 252.78 million [9].

China’s floating population has contributed substantially to increasing prosperity in coastal areas [10] but find it difficult to integrate into urban communities, conferring substantial disadvantage. Firstly, they are less educated than the urban population and few have received work-skills training. Consequently, they typically undertake low-paying and manual jobs, which urban residents are less willing to undertake, in labour-intensive industries such as manufacturing, transportation, construction and services. Secondly and importantly, members of the floating population do not have local household registration (Hukou) which is a record, with information such as name, parents, spouse, and date of birth, in the system of household registration required by laws in China and officially identifies a person as a resident of an area [11]. Thus, they are not entitled to certain local government benefits. This results in daunting problems, such as being unable to use local health insurance, apply for public rental housing, access certain employment opportunities, register for the local pension plans or enrol their children in high-quality local schools [7, 12].

Consistent with these disadvantages, smoking prevalence among male China’s floating population is disproportionately high, although just like the general population, few women from this group smoke [13]. The prevalence rate among male floating population is reported as 54.82%, with smokers consuming an average 18.91 cigarettes daily, far exceeding the general population average, 0.76 , moving to cities does not reduce smoking prevalence or intensity in the floating population: most smokers (69.83%) reported consuming the same amount in their new homes, or even more (27.17%), with only 3.0% smoking less [14]. It is not evident how to reduce the prevalence and intensity of smoking among China’s floating population smokers [15]. Although the magnitude of the problem is known, little is known about the floating population’s understanding, attitudes, social context or behaviours in relation to smoking. Such understanding is necessary to developing effective smoking cessation strategies for this population.

This qualitative study aims, therefore, to describe the reasons for smoking among the floating population in Shanghai, one of the China’s densest floating population concentrations, and why it is so hard for them to stop smoking. This will inform decision-making about whether to develop new approaches or to use or adapt existing interventions.

## Methods

### Research site and sample

This study was conducted in Shanghai, which is one of China’s largest cities and economic centres. Shanghai’s rapid economic development has resulted in increased labour demand, mostly satisfied by the migration from rural areas [16]. The number of inhabitants with Hukou was 14.25 million, while the number of floating population residents reached 9.9 million, which make Shanghai one densest floating population concentration of China [17].

Because Shanghai’s floating population tends to live outside the mainstream, it was appropriate to seek a convenience sample of participants for this study. Specific job sites were targeted (restaurants, hotels, and food markets), where most of the floating population works. Neighbourhood representatives and healthcare providers from local communities facilitated recruitment, contacting job managers by mail or telephone to invite them to participate. Members of the floating population who were current smokers were eligible to participate. Current smoking was defined as either daily or occasional (less than daily) consumption of manufactured or hand-rolled cigarettes. Six focus groups containing a total of 39 male floating population smokers were formed from three worksites (one restaurant one hotel and one food market), with two groups in each worksite. Participants’ socio-demographic characteristics and smoking behaviour are summarised in Table 1. The majority (87%) were younger than 50 years old and only 7.7% had some college or above education. In terms of smoking behavior, more than half smoked more than ten cigarettes per day, while only five out of 39 participants smoked occasionally.

**Table 1:** **Demographic characteristics and smoking behaviour of 39 participants**

	Num (%)
**Marital status**	
Single	13 (33.3)
Married	24 (61.6)
Divorced/windowed/other	2 (5.1)
**Age range (years)**	
18-29	19 (48.7)
30-49	15 (38.5)
50 or older	5 (12.8)
**Education**	
Elementary school or below	7 (17.9)
Junior high school	21 (53.9)
Senior high school	8 (20.5)
Some college and above	3 (7.7)
**Years living in Shanghai (years)**	
Less than 1	6 (15.4)
1-5	12 (30.8)
6-10	14 (35.9)
11 or more	7 (17.9)
**Smoking status**	
Occasionally	5 (12.8)
1-10 cigarettes	12 (30.8)
11-20 cigarettes	9 (23.1)
21 and more	13 (33.3)
**Smoking initiation age (years)**	
Less than 18	11 (28.2)
18-29	23 (60.0)
30 or older	5 (12.8)

Data were analysed after each focus group session with saturation reached after the fifth session. That is, no new themes emerged in the last focus group and there was, therefore, no further recruitment. This study was approved by the Xuhui Health Ethic Committee in Shanghai, China (Protocol number #2013-08).

#### Interview guide

An interview protocol for conducting the focus groups was developed based on the Health Belief Model [18] and the Theory of Planned Behaviour [19], both widely used to explain and predict smoking behaviour [20–23]. The guide covered the following topics: l) time and place of smoking; 2) reasons for smoking; 3) understanding of the health risks of smoking; 5) beliefs about how stopping smoking would improve health; 6) confidence in ability to stop smoking; 7) what significant others think about smoking; 8) contextual factors that promote or inhibit smoking (i.e., social, cultural, economic, political); 9) previous attempts and future intentions to stop, and 10) any other issues participants wished to discuss.

#### Procedure

Drs Wang and Cui (two of the authors) conducted all six focus groups, each containing six or seven voluntary participants and lasting approximately 70–90 minutes. Groups were held in the meeting or lobby room of their worksites in June of 2013. Each participant was assured of his confidentiality in the study, provided informed written consent and completed a demographic questionnaire before the interview started. While participation was voluntary, they received small incentives, such as a notebook or ball-point pen (fair market value US $1), following their participation.

### Data analysis

All focus groups were audiotaped and transcribed in Chinese. Qualitative content analysis was conducted, which could treat materials consistently, ensure that data logic matches argument, provide some reliability and validity checks, help reader evaluate the results, and make your argument persuasive [24]. Drs Cui and Zhang each independently coded one focus group transcript, applying one or more descriptive codes to chunks of text representing each participant’s contributions. They then compared codes, reconciled differences and finalised an agreed coding scheme. Next, Dr Wang applied the coding scheme to all of the transcripts, noting additional emerging codes, which were refined in discussion with Drs Cui and Zhang. Through discussion, the codes were organised into six themes: (i) health risks associated with smoking; (ii) perceptions of smoking as an addiction and habit; (iii) smoking as part of the identity of the floating population; (iv) smoking as part of social participation; (v) smoke-free workplaces; and (vi) stopping smoking. These themes all were raised by all six focus groups. In order to illustrate each theme, representative or powerful quotations were selected and are presented in the results section.

## Results

### Health risks associated with smoking

Most participants knew that the smoking is risky for health and said that smoking had affected their health in some way.

“*I’m always coughing up phlegm. If I walk a long way, I get out of breath. I think this must be because of smoking*.” (food market, 57 years).

However, most did not know why smoking is a health risk. Many thought that smoking would not affect their health if they smoked few cigarettes or stopped in the future.

*“I have heard that smoking causes all kinds of bad diseases, like lung cancer. But I don’t worry too much about it, because I also heard most smokers don’t get lung cancer before they die. I would quit smoking before I get some diseases, therefore smoking would not affect my health.” (food market, 36 years).*

Participants were asked what substances are found in cigarettes, and many of them said they did not know. Few knew the effects of nicotine and tar thinking that modern low-tar cigarettes are less harmful than higher-tar cigarettes.

*“I would like to choose the low-tar cigarettes. I think that modern low-tar cigarettes are less harmful than higher-tar cigarettes because tar are marked on the cigarette boxes.” (restaurant, 32 years).*

*“If smoking is harmful for our health, why doesn’t our country stop the production and sale of cigarettes.” (hotel, 25 years).*

### Perception of smoking as an addiction and habit

Many participants believe smoking is an addiction and described that they could not be separated from smoking, and smoking has been like their friend and a part of life.

*“I always smoke, and I get nervey if I don’t have a cigarette.” (hotel, 22 years).*

*“I always smoke when I wake up and after lunch and supper – that’s become my habit.” (food market, 39 years).*

Participants said that ‘floating’ and living away from family made them feel lonely or isolated, which made them smoke more.

*“When I get home from work, I stay indoors alone because all my family is in my hometown. Smoking helps me feel less lonely and upset.” (restaurant, 32 years).*

Some participants that have been smoking for many years think that they don’t need to change this habit.

*“Smoking is my only fun. I don’t like watching TV, I don’t like drinking – I just like smoking.” (restaurant, 20 years).*

### Smoking as part of the identity of the floating population

Participants spoke about smoking as part of identifying as a member of the floating population. Some participants felt discriminated against because of their smoking, saying that made them more likely to continue to smoke to relieve the stress the discrimination caused. Others saw smoking as their right.

*“Some people from Shanghai say ‘these are outsiders’ when they see us smoking. That means only outsiders smoke, and no Shanghai citizens smoke.” (hotel, 33 years).*

*“If people from Shanghai can smoke, so can we.” (food market, 41 years).*

*“We should also have the right to smoke to make friends. We shouldn’t be deprived of this right just because we are outsiders.” (restaurant, 24 years).*

*“We know smoking in public gives outsiders a bad name, but smoking sometimes helps us make friends, and sometimes we can’t control our cravings for a cigarette.” (restaurant, 29 years).*

### Smoking as part of social participation

Smoking was associated with socialising, sharing and male identity. Participants said that they always smoked when they were with friends, inside and outside work. Handing out cigarettes is very popular among friends or in a social setting. Sharing cigarettes and smoking together is seen as a way to enhance friendships and make more friends. Further, people from the floating population live and work together and, being friends, are inclined to share with each other. This includes sharing cigarettes.

*“When I make a new friend, the first thing I do is offer him a cigarette. If I didn’t do that, my friends would think I’m not generous.” (restaurant, 21 years).*

*“When my friends and I are together, they say ‘come on, let's have a cigarette’ and then they’ll offer me a cigarette. I have to take it and smoke, otherwise they’d think I’m not their friend. Then I have to hand around my cigarettes. As a result, we all smoke more cigarettes.” (food market, 39 years).*

Appearances were important for participants, and their image depended on the price of their cigarettes. They thought that they could win the respect of their friends by smoking expensive cigarettes.

*“I smoke cheap cigarettes while I am alone but I smoke expensive cigarettes when I’m with a friend, or handing cigarettes around to my friends.” (hotel, 39 years).*

*“If you smoke and hand out cheap cigarettes, others think you’re stingy. Nobody would do business with you.” (food market, 31 years).*

### Smoke-free workplace

Most participants said that, if their workplaces were smoke-free, they would comply with these regulations and that this could help reduce their smoking (though nobody thought that such polices would help them completely quit).

*“I don’t smoke at work because my boss bans smoking in all the enclosed areas in our restaurant. I’d be punished or lose my job if I smoked in front of guests.” (restaurant, 28 years).*

*“Smoke-free regulations in my workplace would be good for me and for the customers. They wouldn’t be harmed by second-hand smoke in this market [if smoking were banned]. Some customers don’t like the smell of cigarettes.” (food market, 52 years).*

*“If I smoked in front of my customers, they’d think that we’re no good. I wouldn’t smoke in front of my customers whether or not smoking was banned.” (restaurant, 30 years).*

Some participants said that anti-smoking regulations would only be effective during working hours, and after work they would start smoking again. Workplace smoking bans could not control their smoking outside the workplace.

### Stopping smoking

Participants identified positive and negative attitudes toward stopping smoking. Many respondents reported that quitting smoking would improve their health, prevent them from developing diseases and save them money.

*“It is very hard for us to earn money. Smoking uses up so much of my money that I never have any money left. If I stopped smoking, I’d save this money. Everyone knows that stopping smoking can save money, but it is difficult to quit.” (food market, 36 years).*

Meanwhile, some participants reported that stopping smoking would make others think that they were not ‘real men’. They were also afraid that they would lose their friends and business if they quit smoking.

*“Lots of people think men are supposed to smoke and drink. If I didn’t smoke, they’d say that I am not a man. If I stopped smoking and handing out cigarettes, how could I make friends and do business?” (hotel, 25 years).*

Some participants reported that they had tried to quit smoking but everyone said that they had not received behavioral or pharmacological support from local health professionals. They though that stopping smoking was very hard without help. As members of the floating population, they find it difficult to access local health service for quitting.

*“I want to give up smoking but I can’t.” (restaurant, 31 years).*

*“We’re not natives of Shanghai so we don’t have health insurance. Medical care is so expensive, we’re scared of getting sick.” (food market, 41 years).*

## Discussion

We found that floating population smokers were generally aware that smoking is a health risk but that they had little detailed knowledge. Both habit and social participation were strong drivers of their smoking and smoking was regarded as a core component of their identity by the urban residents. They thought that workplace smoking bans could help make them aware of the need to stop smoking as well as reduce their cigarette consumption during working hours (though not outside of these hours). They recognised the need for professional help to attempt quitting but nobody received any such support.

Our findings are consistent with previous research showing that smoking is socially, economically and culturally rooted in China [6, 25, 26]. As for other Chinese people, smoking among the floating population is not only an addiction and a habit, but also a social activity. Awareness of the health risks of smoking is low in China [27], and we found it to be especially low among the floating population smokers in our study. Their marginalisation and poor access to services and information mean that they are vulnerable to misleading information about smoking. For example, some participants thought it would be better for their health to switch from high tar cigarettes to low tar ones.

Handing out cigarettes was very popular among floating population smokers, and their smoking was often strongly influenced by their peers and their identity as members of the floating population. Participants stated that they had often felt under pressure to accept cigarettes from others as well as handing out their own cigarettes to others. Buying expensive brands of cigarettes appears to be an important part of maintaining a desirable image, even though it frequently leaves them without money. Handing out cigarettes thus plays a vital role in managing and enhancing interpersonal relationships, with the negative consequence of increasing the consumption of cigarettes. Floating population smokers are thus in the difficult situation of knowing that smoking is harmful for their health and costing them money they cannot spare; and needing to keep smoking to remain a respected member of the group. Helping floating population smokers to develop the personal skills to resist accepting and handing out cigarettes is necessary. However, it is only likely to be successful if these skills are developed at the same time as effective action is taken to reduce the perceived social necessity to participate in this exchange and to undermine smoking as a core component of floating population smokers’ identity.

The floating population is widely discriminated against in terms of employment, education, health and housing choice in urban China [28]. Some individuals may use smoking as a way to relieve the psychological stress caused by perceived discrimination [29]. In our study, participants stated that their smoking was connected with how Shanghai locals perceive and act towards the floating population. When members of the floating population perceive that they are mistreated or discriminated against, they tend to smoke more, increasing their social marginalisation. Reducing the prevalence and intensity of smoking among the floating population could help reduce discrimination against them, as could programs to help them effectively cope with such discrimination. This could be particularly effective if programs to reduce discrimination against floating population smokers by local residents could be simultaneously implemented.

Although recent tobacco control legislation in China is an important step in reducing population smoking, enforcement appears minimal at present [6]. Our study indicates that some worksites have established and enforced smoke-free regulations and that these regulations are understood, accepted and obeyed. Workplace smoke-free regulations can help employees reduce their cigarette consumption and stop smoking [30], and do appear to be effective among floating population smokers to a point. However, floating population members tend to live and work with one another in a social context that promotes greater cigarette consumption. Interventions that go beyond enforcing the requirements of tobacco control legislation are therefore needed in this population. Individual and group counselling and pharmacological treatment to overcome nicotine addiction (perhaps conveniently and effectively provided in workplace settings) can be effective in helping smokers who want to quit.

Some participants said that they did not have the skills to quit but felt it would be important for them to stop smoking. These smokers who want to quit but lack the necessary behavioural skills are unlikely to stop smoking, in spite of their motivation to do so. Health professionals have a key role to play in encouraging cessation [31] and supporting the development of necessary skills, and could significantly increase the smoker’s chances of being able to stop smoking successfully [32]. However, the socially isolated floating population [28], lacks access to health professionals and receives health services that are far less than their needs. In our study, some participants clearly expressed their need for professional assistance to quit smoking and just as clearly stated that no such assistance was available. As these smokers are isolated from local health services and marginalised socially, it is difficult to access local smoking cessation programs.

Because a person’s smoking influences the smoking behaviour of others nearby in the social network, collective or group-based smoking cessation interventions may be more effective than individual interventions [33], or an essential adjunct. Local health professionals may thus obtain more success if they focus their smoking cessation efforts on the group rather than on individual members of the floating population. Quit smoking groups could be used to conduct a variety of activities, such as: information sharing and lectures on the health hazards of smoking and the benefits of stopping; activities that facilitate smoking behaviour change; group not-yet-ready-to-change counselling sessions; ready-to-change counselling sessions; and identifying resistance behaviours and potential strategies to modify them.

Certain limitations of this study may inhibit our ability to generalise from our findings. Participants in the focus groups were a convenience sample and may not be representative of the entire target population in terms of education, acculturation, literacy levels and other demographic variables. Our findings may therefore not apply to all floating population smokers. Further, participants who volunteer to take part in a discussion about smoking may not be typical of all floating population smokers. Finally and importantly, this study was based on a sample of men and might not apply to the women floating population smokers. Even though female smoking prevalence is low in China, a smoking cessation study among the female floating population smokers is necessary as it cannot be assumed their perceptions are the same as those of their male peers, and their health is just as important. Large-scale quantitative study of men and women floating population smokers, informed by this study, would assist in addressing these weaknesses and help in planning interventions that would be likely to be effective.

## Conclusions

Our findings show that there is a critical need to gain deeper understanding of the experiences and needs of the floating population; and thereby to develop programs for this population and the non-floating population that can successfully promote smoking cessation for all Chinese people, especially this high-risk and deeply disadvantaged group. These programmes should focus on exploring and addressing the social influences which encourage smoking, especially in terms of the exchanging of (expensive) cigarettes as a key social currency. Implementing group workplace-based smoking cessation programs would be well accepted and could thus be particularly effective; and using health professional to provide smoking-related education would help meet floating population smokers’ information needs. Our study takes a positive first step in providing information to help understand the experiences and environment of smoking among Shanghai’s floating population.
